# A Method for Enhancing the Sensing Distance of a Fingerprint Sensor

**DOI:** 10.3390/s17102280

**Published:** 2017-10-07

**Authors:** Kyung-Hoon Song, Jaehuk Choi, Jung-Hoon Chun

**Affiliations:** 1 College of Information and Communication Engineering, Sungkyunkwan University, 2066 Seobu-ro, Jangan-gu, Suwon, Gyeonggi-do 16419, Korea; kh1.song@samsung.com (K.-H.S.); choix215@skku.edu (J.C.); 2 Advanced Component Technology Group, Mobile Communication Division, Samsung Electronics Ltd., 129 Samsung-ro, Yeongtong-gu, Suwon, Gyeonggi-do 16677, Korea

**Keywords:** capacitive fingerprint sensor, fingerprint recognition, fingerprint image enhancement

## Abstract

In this paper, we describe a method for improving the quality of fingerprint images at long sensing distances by reducing the fringe capacitance formed between a pixel and surrounding fingerprint patterns. Air-walls were applied to the glass attached to a fingerprint sensor for reducing the edge capacitance. Fingerprints and air-wall structures were modeled using a three-dimensional capacitance analysis tool. A prototype was fabricated by stacking glass layers with air-walls with a depth of 50 μm and a pitch of 50 μm on a silicon-type capacitive sensor. Using the results of simulations and prototype experiments, we confirmed that the proposed air-wall structure achieved high enough resolution to distinguish 2.5-lp/mm fingerprint patterns at a sensing distance of 300 μm or longer, and its contrast improved from 0.59 to 0.98.

## 1. Introduction

Recently, with the increasing size of mobile payment markets there has been a trend to equip mobile devices with biometrics-based personal authentication services [1,2]. The commonly used biometrics authentication method is fingerprint authentication, which is both convenient and secure. Among a variety of fingerprint sensors such as capacitive, optical, ultrasonic, and thermal sensors, capacitive fingerprint sensors are prevalently used because they are low-cost and can be easily miniaturized. Capacitive fingerprint sensing achieves fingerprint pattern recognition by sensing capacitance changes caused by different distances of the ridge and the valley from the sensor surface [3,4,5,6,7,8,9,10,11]. In addition to the basic recognition performance, mechanical durability must be ensured because sensors are mounted on mobile devices that are exposed to the outside such as a front home button or a rear button. Hence, most of the commercially available capacitive fingerprint sensors have a layer of protective coating on top of an epoxy mold compound (EMC) layer which encapsulates a silicon sensor chip. The thickness of the coating layer is limited to 50–100 µm because of the sensing distance limitation. The coating method has the advantages of high process yield and low unit cost, but the coating is susceptible to scratches owing to insufficient surface hardness. In addition, color reproducibility can be degraded because of the additional coating layer.

Previous circuit-level studies have been conducted on subjects, such as developing a method for improving the signal-to-noise ratio by reducing the parasitic capacitance of pixels [8,9], and developing a method for automatically coping with contaminations of the sensor surface by implementing pixel-by-pixel calibration [8]. In the non-circuit area, research has been conducted to improve the sensing performance by increasing the dielectric constant of the package molding material [12,13] and to spatially confine the electric field to increase the resolution of fingerprint images [14]. There are commercially available sensors that involve a method of stacking relatively durable, high-quality glass on a sensor chip, rather than the aforementioned coating method. However, as the sensing distance still remains at 250 µm or so, a method of laminating a thin glass layer on a sensor chip or mounting a fingerprint sensor under a partially recessed cover glass has been recently introduced. These structures improve hardness and appearance quality compared with the coated sensors, but they also have limitations such as their relative fragility owing to the presence of a thin glass layer, low yield, and high processing cost.

The sensing distance in these sensors is small, because the larger the distance from the sensor surface to the fingerprint, the smaller the difference in capacitance between ridges and valleys. In particular, for sensing distances larger than 300 µm, the difference is dramatically reduced, and the resolution and contrast of fingerprint images are significantly reduced. This primarily occurs because the fringe capacitance between the pixel and the peripheral fingerprint pattern can be larger than the capacitance formed between the pixel and the fingerprint normal to the pixel as the sensing distance increases. In this paper, we propose a method for improving the resolution and contrast of fingerprint images at sensing distances of 300 µm or longer by reducing the edge capacitance. Simulations show that the difference in capacitance between ridges and valleys increases while using air-walls in the passivation layer between the pixels. In the prototype validation, the resolution of a captured fingerprint image was higher than 2.5 lp/mm, and the contrast improved from 0.59 to 0.98.

## 2. Method and Simulation

### 2.1. Method

**Figure 1:**
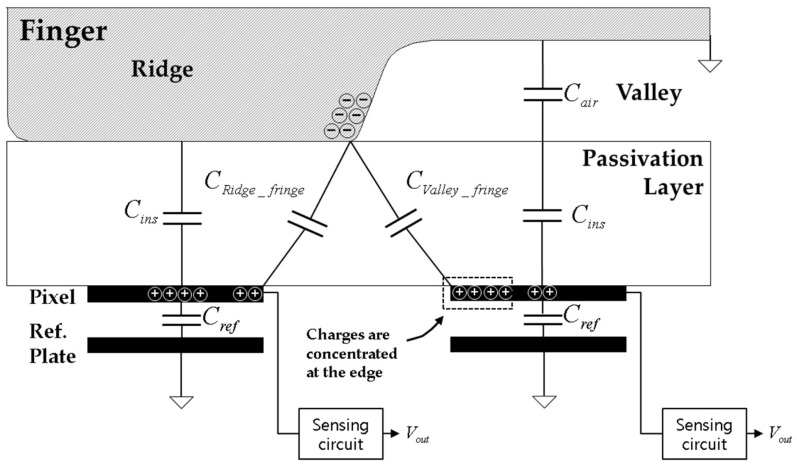
Capacitive fingerprint sensor.

In this analytical study, several layers from the sensor surface to the fingerprint surface were simplified as one passivation layer. Figure 1 shows the structure of a general capacitive fingerprint sensor. The ridges touch the surface of the passivation layer and the valleys float without touching the surface. Since the ridges and valleys differ in their distance from pixels and in their dielectric constants, a difference in capacitance is caused. The capacitive fingerprint sensor senses the difference and recognizes the pattern of the sensed fingerprint.

**Figure 2:**
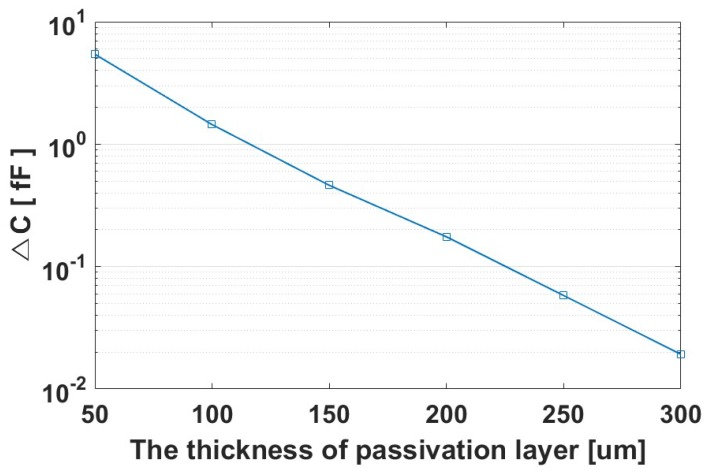
Δ*C* vs. the thickness of the passivation layer.

**Figure 3:**
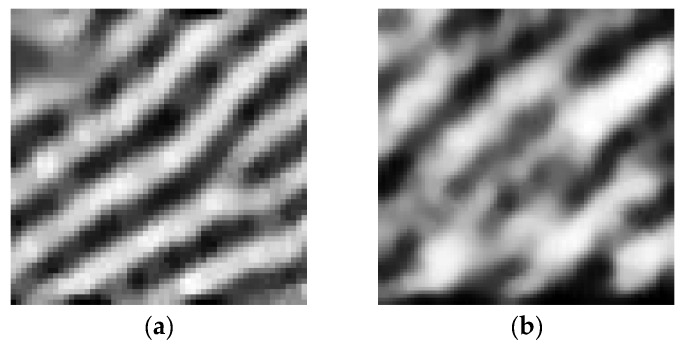
Fingerprint images for different sensing distances. (**a**) Image for a 250-µm-thick passivation layer; (**b**) Image for a 300-µm-thick passivation layer.

The amplitude of the input signal is determined by the capacitance difference, Δ*C*, between the ridges and the valleys. The capacitance difference Δ*C* is given by Equation (1). In this equation *C_Ridge_total_* is the capacitance between the pixel under a ridge and the ridge, and it is the sum of *C_ins_* and *C_Ridge_fringe_*; *C_ins_* is the capacitance of the passivation layer; *C_Ridge_fringe_* is the fringe capacitance formed between the pixel and its adjacent ridge; *C_Valley_total_* is the capacitance between a valley and the pixel under the valley, and it is the sum of *C_Valley_fringe_* and series capacitance of *C_ins_* and *C_air_*; *C_Valley_fringe_* is the fringe capacitance formed between the pixel under the valley and its adjacent ridge; *C_air_* is the capacitance formed between the surface of the passivation layer and the valley. As the passivation layer’s thickness increases, Δ*C* decreases, as shown in Figure 2, and image quality deteriorates as shown in Figure 3.

(1) $$\begin{array}{ll} △C & =\;C_{Ridge\_total}-C_{Valley\_total}\\ & =\;(C_{ins}+\;C_{Ridge\_fringe})\;-(\frac{C_{air}C_{ins}}{C_{air}+C_{ins}}\;+C_{Valley\_fringe})\\ & =\;\frac{C_{ins}{}^{2}}{C_{air}+C_{ins}}\;+(C_{Ridge\_fringe}-C_{Valley\_fringe}) \end{array}$$

**Figure 4:**
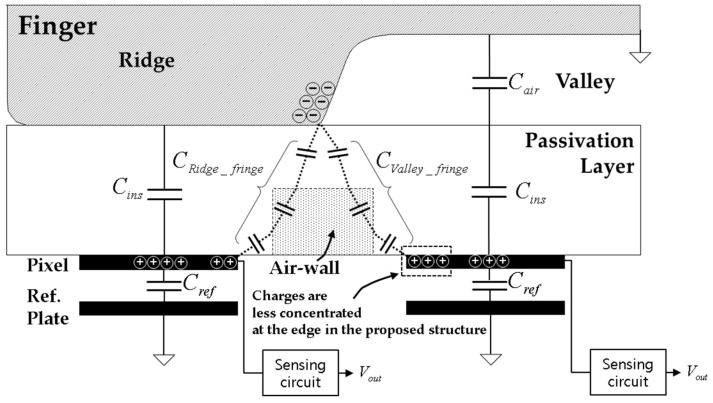
Proposed structure with an air-wall in the passivation layer.

In this study, we propose a method for increasing Δ*C* by using an air-wall in the passivation layer, as shown in Figure 4. The air-wall has a lower dielectric constant compared with the other parts of the passivation layer. Due to this relatively low dielectric constant, both *C_Ridge_fringe_* and *C_Valley_fringe_* of the proposed structure are smaller than those of the conventional structure. However, a decrease in *C_Valley_fringe_* is larger than that of *C_Ridge_fringe_* because most of the charges in the pixels under the valleys are concentrated at the edges near adjacent ridges, as illustrated in Figure 1 and Figure 4. As a result, we can achieve a net increase in Δ*C* as described in Equation (1).

### 2.2. Simulation

To predict the increase in Δ*C* and the improvement of image quality incurred by the proposed air-wall structure, we simulated the proposed fingerprint sensor structure.

The geometric characteristics of the fingerprint patterns, width and pitch of ridges and valleys, differ from fingerprint to fingerprint, but in this study we simplified the modeling process by considering average widths of ridges and valleys. For adult males, the ridges and valleys are 300-μm-wide, while for adult females, the width is 200 μm [15]. The average spatial frequencies are 1.67 lp/mm and 2.5 lp/mm, respectively. Because the average spatial frequency for adult females is higher than that of adult males, a spatial frequency of 2.5 lp/mm was used as a criterion for improvements in the fingerprint image quality. The spatial frequency, lp/mm, represents the number of line pairs per a millimeter. Here, a line pair means a pair of one black line and one white line. The depth of the valley was set to 50 μm. When ridges contacted the surface of the passivation layer, it was assumed that the depth of the valley decreased from the initial value of 150 μm (measured value) to about 50 μm, owing to the application pressure. Table 1 lists the dimensions of the fingerprint patterns used in our modeling.

**Figure 5:**
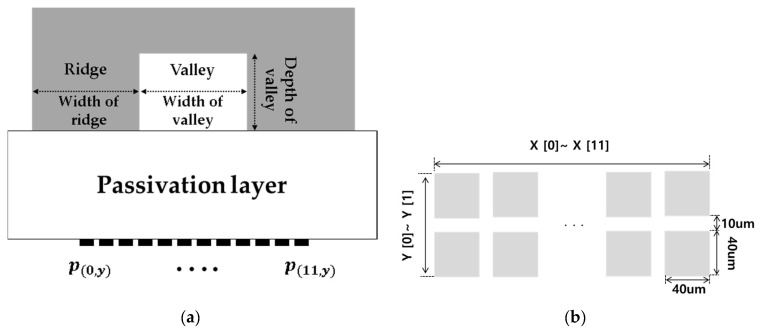
Modeling of a fingerprint, the passivation layer and the array of pixels: (**a**) Cross-sectional view; (**b**) Array of pixels.

**Figure 6:**
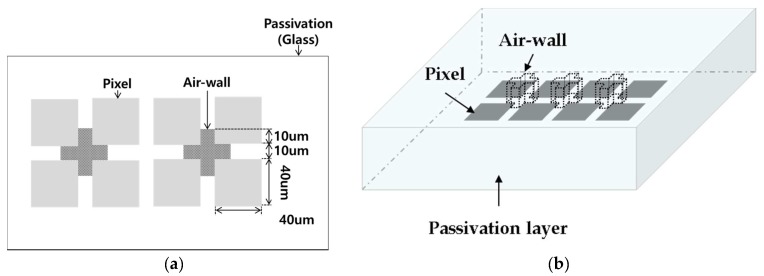
Modeling of the air-walls in the passivation layer: (**a**) top-view; (**b**) three-dimensional view.

Figure 5a shows the cross-sectional view of the model with the geometric characteristics of the above-mentioned fingerprint. The model fingerprint has a center valley surrounded by ridges, and the physical properties were set to those of a perfect electric conductor (PEC). The passivation layer under the fingerprint was glass, with a relative dielectric constant of 8.1. Below the glass layer there was an array of pixels as shown in Figure 5b. The pixels were thin conductors, each pixel was 40 µm × 40 µm and the pitch was 50 μm. Figure 6 schematically shows the air-wall structure. As shown in Figure 6a, the air-wall is cross-shaped, with a length of 10 μm on all sides and a depth of 50 μm. The reason for applying such a cross-shaped air-wall to the corner of the pixel is related to the edge effect of charge. If the air-wall is present on all sides, the overall dielectric constant of the passivation layer is reduced and Δ*C* is reduced. In contrast, in regions of high charge density the proposed air-wall can reduce the fringe capacitance while minimizing the overall dielectric constant reduction of the passivation layer.

In the simulation, the performance was characterized in terms of Δ*C_avg_* and Δ*C_norm_*, which is the average Δ*C_avg_* normalized by the average ridge capacitance as shown in Equation (2). Δ*C_avg_* is the difference between the averaged capacitance of pixels underneath a ridge, *C_average of ridges_*, and that of pixels underneath a valley, *C_average of valleys_*.

(2) $$\Delta C_{norm}=\frac{\Delta C_{avg}}{C_{average\;of\;ridges}}=\frac{C_{average\;of\;ridges}-C_{average\;of\;valleys}}{C_{average\;of\;ridges}}$$

**Figure 7:**
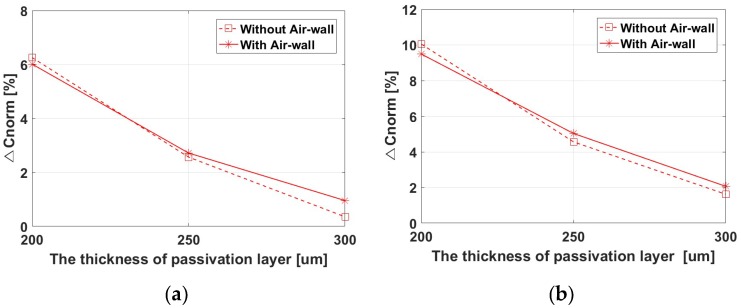
Performance index Δ*C_norm_* vs. thickness of the passivation layer, for the systems with and without an air-wall: (**a**) Fingerprint pattern frequency of 2.5 lp/mm; (**b**) Fingerprint pattern frequency of 1.67 lp/mm.

In Figure 7, the performances of the structures with and without the air-wall simulated by a capacitance extraction tool are compared. Figure 7a shows the dependence of the Δ*C_norm_* on the thickness of the passivation layer when the spatial frequency of the fingerprint pattern is 2.5 lp/mm, corresponding to the average female figure. The Δ*C_norm_* for the system with the air-wall structure was higher by ~1.4% when the thickness of the passivation layer was 300 µm. After converting the capacitance data of the pixel array obtained in the simulation into an 8-bit grayscale image, the image quality according to the thickness of the passivation layer was compared, and the results are listed in Table 2. In the image, white areas correspond to ridges and black areas correspond to valleys. When the thickness of the passivation layer was 300 μm, the contrast was significantly better for the structure with the air-wall. Figure 7b shows the Δ*C_norm_* for the spatial frequency of the fingerprint pattern set to 1.67 lp/mm, corresponding to the average male figure. The Δ*C_norm_* for the system with the air-wall structure improved by ~0.4% when the thickness of the passivation layer was 300 μm. Table 3 compares the converted images. When the thickness of the passivation layer was 300 μm, the contrast was improved by applying the proposed air-wall. Because the distance between a pixel below a valley and its adjacent ridge is shorter at 2.5 lp/mm than 1.67 lp/mm, the contribution of *C_Valley_fringe_* to Δ*C_avg_* at 2.5 lp/mm is relatively high, so image deterioration is worse. Thus, the effect of the air-wall is stronger at higher spatial frequencies. The differences in Figure 7 may look very small. But this small differences can make the signal strength with the proposed air-wall exceed the boundary value for producing distinguishable fingerprint pattern images.

The simulation results are summarized in Table 4. There is no standardized quantitative index for required image quality because the performance differs across sensor systems depending on not only the noise behavior of ADCs and analog front-end (AFE) circuits but also software features such as an image processing and a matching algorithm. However, in capacitive fingerprint sensors, the capacitance variation, Δ*C*, across sensing pixels is recognized as an effective signal, and Δ*C_avg_* can be a useful metric in evaluating the performance of sensing parts when the AFE and ADC circuits are fixed. For example, the matching algorithm in the implemented fingerprint sensor requires ENOB of 3 bits for the ADC when the signal of Δ*C_avg_* is applied. In this work, 8-bit, 10-MHz ADCs with the excitation voltage of 3V is used, and Δ*C_avg_* larger than 0.02 fF is needed to meet the requirement of 3-bit ENOB. At 2.5 lp/mm, when the thickness of the passivation layer is 300 μm, Δ*C_avg_* is improved from 0.003 *fF* to 0.025 *fF* by applying the air-wall.

## 3. Measurement Results

**Figure 8:**
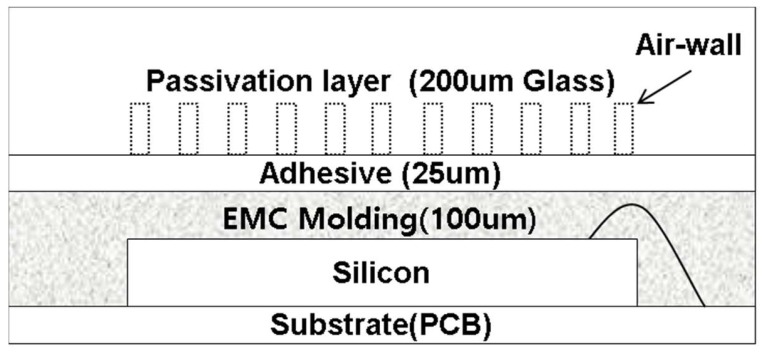
Cross-sectional view of the prototype with the air-wall in the passivation layer.

**Figure 9:**
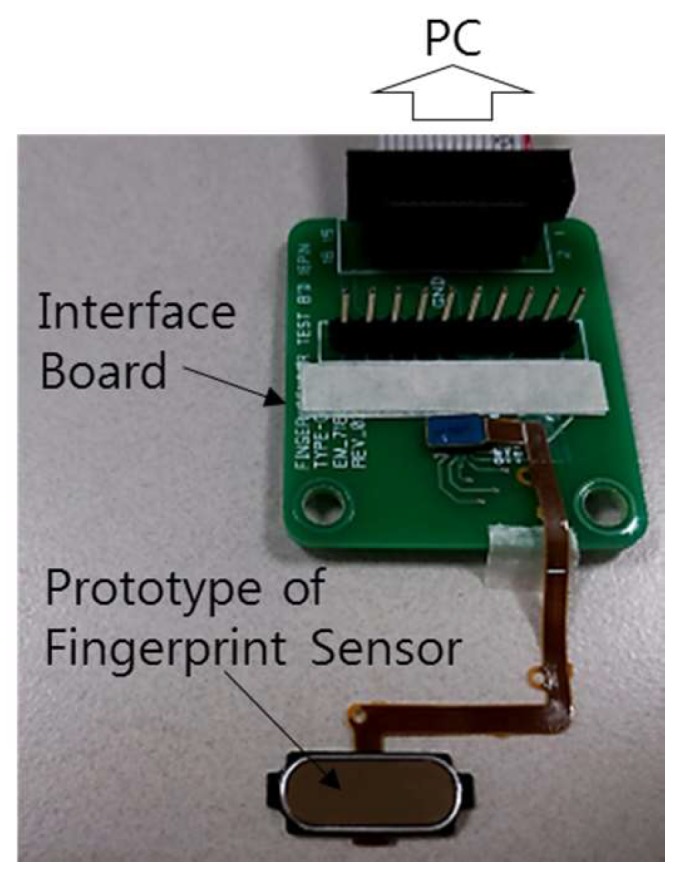
Prototype fingerprint sensor and an interface board.

Figure 8 shows a cross-sectional view of the fabricated prototype sensor. The passivation layer was a thin film glass, with a thickness of 200 μm and a relative dielectric constant of 8.1. The air-wall was laser-processed, with a depth of ~50 μm and a pitch of ~50 μm. Below the thin film glass there was a 25 μm-thick adhesive layer with a relative dielectric constant of 3.5. The fingerprint sensor used in the experiment was a self-capacitive type fingerprint sensor, the resolution was 508 dpi, and the package type was EMC encapsulated on silicon with an array of pixels. There was a 100 μm-thick EMC layer between the silicon layer and the adhesive layer, so the total sensing distance was about 325 μm. In addition, a prototype without the air-wall was fabricated for performance comparison. Figure 9 is a picture of the prototype fingerprint sensor and an interface board.

**Figure 10:**
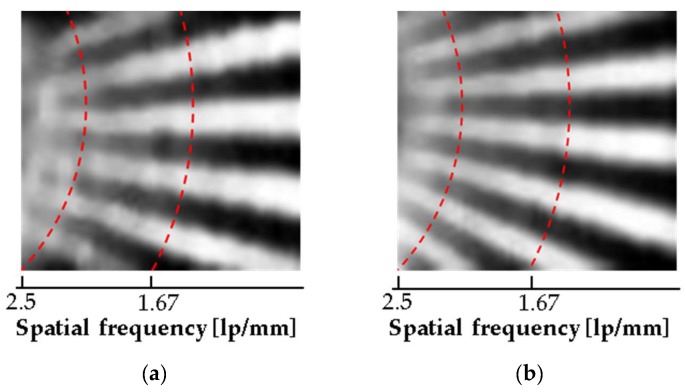
Comparison of image resolutions, for images captured for the pattern rubber with and without an air-wall: (**a**) without an air-wall in the passivation layer, (**b**) with an air-wall in the passivation layer.

Figure 10 shows comparative data of images captured with a conductive pattern rubber. Figure 10a, which is the image captured by the conventional sensor without the air-wall, shows blurry boundaries between ridges and valleys at a spatial frequency of 2.5 lp/mm. However, the proposed sensor with the air-wall could clearly distinguish ridges and valleys at spatial frequencies even higher than 2.5 lp/mm.

**Figure 11:**
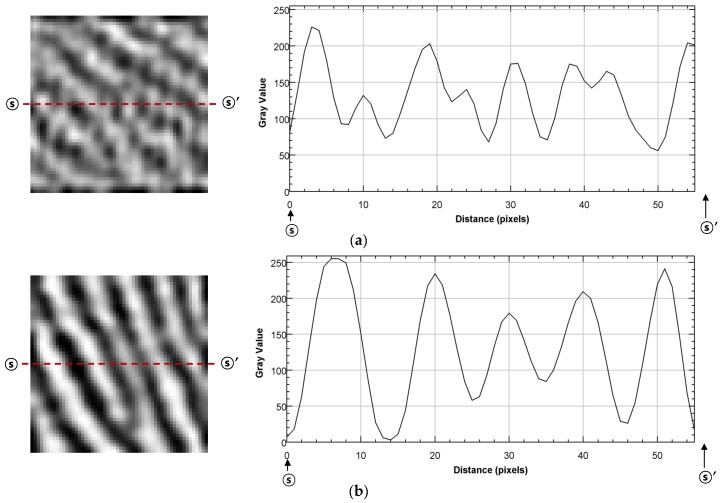
Comparison of gray values of images captured for a real finger, for the systems with and without an air-wall: (**a**) without an air-wall in the passivation layer; (**b**) with an air-wall in the passivation layer.

Figure 11 shows comparative data of images captured with a real fingerprint pattern. To make the frequency of the fingerprint pattern as close as possible to 2.5 lp/mm, the narrow part of the valley was captured. Figure 11a shows the fingerprint image and the gray value graph obtained using the prototype without the air-wall. Figure 11b shows the fingerprint image and the gray value graph obtained using the prototype with the air-wall. While comparing the gray value graphs, it is evident that the ridge and the valley are clearly distinguished in the prototype with the proposed air-wall. The contrast was 0.59 for the system without the air-wall, and improved to 0.98 for the system with the air-wall. The contrast was calculated using the maximal and minimal gray values, as shown in Equation (3).

(3) $$\mathrm{Contrast}=\frac{\max\;\mathrm{of}\;\mathrm{gray}\;\mathrm{value}\;-\;\min\mathrm{of}\;\mathrm{gray}\;\mathrm{value}}{\max\;\mathrm{of}\;\mathrm{gray}\;\mathrm{value}\;+\;\min\mathrm{of}\;\mathrm{gray}\;\mathrm{value}}$$

## 4. Conclusions

In this paper, we proposed a method for increasing the sensing distance of fingerprint sensors by increasing the effective input signal, by reducing the edge capacitance formed between pixels and ridges of adjacent fingerprints by incorporating air-walls in the passivation layer of the fingerprint sensor. Based on the analysis of fingerprint characteristics, fingerprints were modeled. The amount of edge capacitance reduced by the proposed air-wall and the improvement of fingerprint images’ quality were predicted through simulations. In the prototype experiments, the resolution of the fingerprint sensor improved to higher than 2.5 lp/mm and the contrast improved from 0.59 to 0.98 at the sensing distance of 300 µm or longer.

## Figures and Tables

**Table 1:** Dimensions of the model fingerprint.

Group	Width of Ridge	Width of Valley	Depth of Valley
Male adult	300 µm	300 µm	50 µm
Female adult	200 µm	200 µm	50 µm

**Table 2:** Image quality comparison, for the systems with and without an air-wall, for the spatial frequency of 2.5 lp/mm.

Passivation Thickness (µm)	Without Air-Wall	With Air-Wall
200	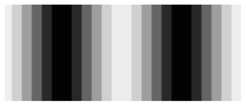	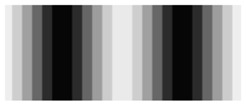
250	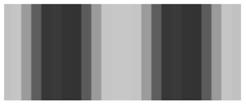	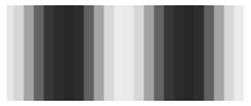
300	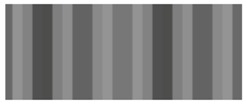	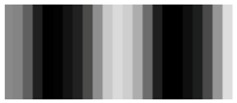

**Table 3:** Image quality comparison, for the system with and without an air-wall, for the spatial frequency of 1.67 lp/mm.

Passivation Thickness (µm)	Without Air-Wall	With Air-Wall
**200**	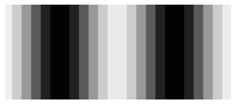	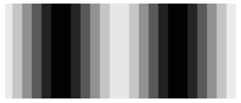
**250**	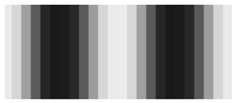	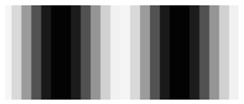
**300**	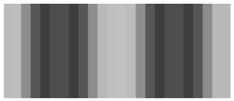	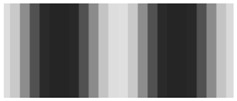

**Table 4:** Summary of the simulation results.

Passivation Thickness		200 µm		250 µm		300 µm	
Spatial Frequency (lp/mm)		Without Air-Wall	With Air-Wall	Without Air-Wall	With Air-Wall	Without Air-Wall	With Air-Wall
**2.5**	***ΔC****_norm_* **[%]**	**7.92**	**6.26**	**2.57**	**3.99**	**0.21**	**1.63**
	***ΔC****_avg_* **[fF]**	**0.169**	**0.147**	**0.049**	**0.72**	**0.003**	**0.025**
**1.67**	***ΔC****_norm_* **[%]**	**10.05**	**9.49**	**4.56**	**5.03**	**1.62**	**2.07**
	***ΔC****_avg_* **[fF]**	**0.236**	**0.198**	**0.086**	**0.090**	**0.027**	**0.032**
